# Enhancing stability and safety of chimeric peptidoglycan hydrolases by linker engineering

**DOI:** 10.1007/s00253-025-13651-7

**Published:** 2026-01-12

**Authors:** Paweł Mitkowski, Elżbieta Jagielska, Małgorzata Korzeniowska nee Wiweger, Marzena Nowacka, Morten Kjos, Christian Kranjec, Izabela Sabała

**Affiliations:** 1 Laboratory of Protein Engineering, Mossakowski Medical Research Institute Polish Academy of Sciences, Warsaw, Poland; 2 Laboratory of Molecular Oncology and Innovative Therapies, Military Institute of Medicine, National Research Institute, Warsaw, Poland; 3 Faculty of Chemistry, Biotechnology and Food Science, Norwegian University of Life Sciences, Ås, Norway

**Keywords:** Bacteriolytic enzyme, *Enterococcus faecalis*, *Staphylococcus aureus*, Linker, Protein engineering, Peptidoglycan hydrolase

## Abstract

**Abstract:**

Spread of antimicrobial resistance and lack of new antibiotics have brought attention to alternative strategies of combating pathogenic bacteria. One of these strategies takes advantage of the bacteriolytic activity of peptidoglycan hydrolases. The enzymes allow efficient elimination of pathogenic bacteria while preserving the natural microflora. Such enzymes must meet specific criteria of activity, stability, and safety to become efficient enzybiotics. In our previous work (10.1128/spectrum.03546-23), we have created three chimeric enzymes and demonstrated their high efficacy in the elimination of *Enterococcus faecalis* and *Staphylococcus aureus*. In this work, we investigated and addressed issues related to the stability and safety of these enzymes. To improve the stability, we engineered the linkers and optimized storage conditions. Moreover, we demonstrated that such enzymes do not have any cytotoxic effects on eukaryotic cells, *Danio rerio* or *Galleria mellonella*. We also investigated the prevalence of resistance development, a particularly important feature for new antimicrobials. In conclusion, we here propose efficient, safe, and stable chimeric enzybiotics to eliminate *E. faecalis* and *S. aureus*.

**Key points:**

• *Optimized linker design enhances enzyme stability.*

• *Generated chimeric lysins do not display cytotoxicity.*

• *Chimeras with minimal risk of resistance development were selected.*

**Supplementary Information:**

The online version contains supplementary material available at 10.1007/s00253-025-13651-7.

## Introduction

Antimicrobial resistance (AMR) stands as one of the most pressing global health challenges of our time. The emergence and spread of antibiotic-resistant pathogens have rendered many conventional antibiotics obsolete. Despite concerted efforts to combat this crisis, the adaptability of microbes continues to outpace our existing arsenal of antimicrobial agents. To mitigate the escalating threat of AMR and safeguard public health, there is an urgent need to explore and develop novel therapeutic approaches that could replace and/or complement traditional antibiotic treatments. Alternative strategies, ranging from bacteriophage therapy (Kushwaha et al. 2024; Pal et al. 2024) and immunotherapeutic interventions (Qadri et al. 2023) to antimicrobial peptides (Heilbronner et al. 2021; Fernandes and Jobby 2022) and bacteriolytic enzymes (Nelson et al. 2012; Murray et al. 2021; Kocot et al. 2023), offer promising avenues for combating resistant pathogens while minimizing selective pressure for further resistance development.

Among these alternatives, there is a rising interest in the application of bacteriolytic enzymes, such as bacterial autolysins and endolysins derived from bacteriophages. Bacteriolytic enzymes exhibit potent antimicrobial activity by targeting and cleaving specific components of the bacterial cell wall, leading to rapid bacterial lysis and death (Nelson et al. 2012). Unlike conventional antibiotics, bacteriolytic enzymes have a precisely defined, usually narrow spectrum of activity, targeting only specific bacterial species or strains, thus minimizing disruption to the beneficial microbiota. They are also considered antimicrobials with a low prevalence of resistance development, a feature which is particularly relevant in light of the current antimicrobial resistance crisis (Eichenseher et al. 2022). Moreover, these enzymes hold the advantage of synergistic activity when used in combination with conventional antibiotics, enhancing the efficacy of antimicrobial therapy and overcoming resistance mechanisms employed by bacteria (Hong et al. 2022).

The structures, number of domains, specificity of the peptidoglycan hydrolases as well as their functions within the cell vary considerably (Vermassen et al. 2019). Typically, they comprise binding and catalytic domains and display a wide range of features with regard to specificity, efficacy, and adaptation to certain environmental conditions. Often, their activity in biological media, like in serum or milk, is compromised (Verbree et al. 2018; Keller et al. 2022). This structural diversity facilitates the engineering of novel chimeric enzymes with improved properties compared to their parental enzymes. Such properties include, for example, expanded specificity or activity under novel environmental conditions and complex biological media. It has previously been demonstrated in our laboratory that by adding a peptidoglycan binding domain to the M23 catalytic domain, we could expand the tolerance of the chimeric enzymes to a broad spectrum of ionic and pH conditions (Jagielska et al. 2016; Mitkowski et al. 2024; Kaus-Drobek et al. 2025).

EnpA is a large multidomain protein of prophage origin present in *Enterococcus faecalis* genomes. The protein consists of a phage-related tail protein domain, a murein D,D-endopeptidase MepM, a murein hydrolase activator NlpD containing a LysM domain, a peptidase M23, and a lytic transglycosylase-like domain (Reste De Roca et al. 2010). M23 domains are zinc metalloendopeptidases that cleave bonds in peptidoglycan (Razew et al. 2022). In our previous studies, we have demonstrated that the isolated M23 domain of EnpA (EnpA_CD_) displays potent lytic activity against a wide range of bacterial species, including its host *E*. *faecalis* as well as some staphylococci and streptococci, but its activity was limited to low ionic strength conditions (Małecki et al. 2021). We have further improved the enzyme properties by fusing the catalytic domain to three different SH3b cell wall binding domains and thereby gained an improved efficacy of the chimeric enzymes with activities in a broader spectrum of pH and ionic conditions (Mitkowski et al. 2024). Such chimeric enzymes can find application in the prevention or treatment of infectious diseases caused by enterococci and staphylococci. However, the limited stability of the generated chimeric enzymes confines this potential. Thus, in this work, we have further engineered the enzymes to improve their stability, tested their efficacy in complex biological media, defined conditions for long-term storage, and proved their safety in three different models. Moreover, we have also tested the prevalence of resistance induction and showed that our bacteriolytic enzymes can be combined with other natural antimicrobials, namely bacteriocins.

## Materials and methods

### Engineering of chimeric enzymes with modified linker

The linkers of the initial chimeras were shortened or mutated using the PIPE method and point mutagenesis, respectively. Previously generated expression constructs were used as templates (Mitkowski et al. 2024). For this purpose, special pairs of primers have been prepared (Table 1). The PCR program: 98 °C - 30 s, (98 °C - 7 s, 55 °C - 20 s, 72 °C - 90 s) × 35 cycles, 72 °C - 300 s, 18 °C - hold was used to amplify the DNA fragments. The PCR products were treated with DpnI (EureX) for 1 h to remove the DNA template. The constructs obtained were sequenced.

**Table 1:** Primers used in linker engineering

Protein variant	Abbr	5′->3′ sequence	
EL short 1	EL S1	Forward	GATACTTATGGTGGTACAGTAACTCCAACGCCG
		Reverse	TGTACCACCATAAGTATCAGGATTTACGTGTTG
EL short 2	EL S2	Forward	GATACTTATACAGTAACTCCAACGCCGAATACA
		Reverse	AGTTACTGTATAAGTATCAGGATTTACGTGTTG
EL mutant	EL M	Forward	GAAAAGCAGCTTCTACAGTAACTCCAAC
		Reverse	GTTACTGTAGAAGCTGCTTTTCCATATC
EP short 1	EP S1	Forward	GATACTTATAGTGGTAAGAACCCATCTACTTCT
		Reverse	CTTACCACTATAAGTATCAGGATTTACGTGTTG
EP short 2	EP S2	Forward	GATACTTATAAGAACCCATCTACTTCTAACGGA
		Reverse	TGGGTTCTTATAAGTATCAGGATTTACGTGTTG
ES short 1	ES S1	Forward	GATACTTATTCTGGTTATACTCCGCCGGTTAAT
		Reverse	ATAACCAGAATAAGTATCAGGATTTACGTGTTG
ES short 2	ES S2	Forward	GATACTTATTATACTCCGCCGGTTAATCGCGTA
		Reverse	CGGAGTATAATAAGTATCAGGATTTACGTGTTG

### Proteins expression

Sequence-confirmed plasmids were used to transform *E. coli* BL21 (DE3). Protein expression was carried out in autoinduction medium LB (AIM-LB) with 50 mg/l ampicillin at 25 °C overnight with shaking at 180 rpm.

### Proteins purification (before buffer optimization)

The cells were centrifuged at 4000 rcf for 10 min and then resuspended in buffer A (50 mM Tris–HCl pH 8.0, 500 mM NaCl, 20 mM imidazole, 10% glycerol). Bacteria suspension was sonicated on ice for 5 min (cycle: 15 s work, 60 s rest) and centrifuged at 20,000 rcf for 40 min at 4 °C. The supernatant was loaded onto a HisTrap HP 5 ml column (GE Healthcare), washed in buffer A, and gradually eluted to buffer B (20 mM Tris–HCl pH 8.0, 200 mM NaCl, 500 mM imidazole; 10% glycerol). The obtained protein solution was dialyzed overnight in the presence of TEV protease at room temperature in 20 mM Tris–HCl pH 8.0, 200 mM NaCl, 10% glycerol. The protein solution was reapplied to the HisTrap FF 1 ml column, and the enzyme without HisTag was collected in the flow-through. Fractions containing overexpressed protein were concentrated and loaded onto the 26/600 Superdex 75 prep grade, size-exclusion chromatography column (GE Healthcare). The separation was performed in buffer B without imidazole. The peak fractions were analyzed by SDS-PAGE. Pure protein was concentrated, flash frozen, and stored at − 80 °C.

### Protein purification (after buffer optimization)

Bacteria with overexpressed protein were suspended in 50 mM HEPES pH 7.5, 1 M NaCl, 10% glycerol, 20 mM imidazole. The sample was sonicated on ice for 5 min (cycle: 15 s of work, 60 s of rest), centrifuged (20,000 rcf, 30 min, 4 °C), and the supernatant with soluble protein was applied to the HisTrap FF 1 ml column (Cytiva). The column was washed with a buffer used for sonication. The recombinant protein was eluted with 20 mM HEPES pH 7.5, 0.4 M NaCl, 10% glycerol, 500 mM imidazole. The protein solution was dialyzed overnight together with TEV protease at room temperature in 20 mM HEPES pH 7.5; 400 mM NaCl, 10% glycerol (TEV protease was added to remove HisTag). The protein solution was reapplied to the HisTrap FF 1 ml column, and the enzyme without HisTag was in the flow through (TEV protease and non-cleaved enzyme remained on the column). The last step was size exclusion chromatography carried out in a dialysis buffer, using a HiLoad 16/600 Superdex 75 pg column (Cytiva). The pure protein was flash frozen in liquid nitrogen and stored at −80 °C for further tests.

### Protein stability in different buffers

One hundred microliters of purified protein (5 µg/ml) in 20 mM HEPES pH 7.5, 400 mM NaCl, 10% glycerol was mixed with 400 µl of the following buffers: 50 mM citric acid pH 3.2, 500 mM NaCl; 50 mM Na^+^/K^+^ phosphate pH 5.0, 500 mM NaCl; 50 mM ADA pH 6.5, 500 mM NaCl; 50 mM HEPES pH 7.5, 500 mM NaCl; 50 mM Tris–HCl pH 8.5, 500 mM NaCl; 50 mM CAPS pH 10, 500 mM NaCl; 20 mM Tris pH 8.0, 200 mM NaCl, 10% glycerol, and stored at 4 °C or room temperature for 4 weeks.

### Enzyme activity—turbidity reduction assay

The bacteria from glycerol stock were streaked on TSB-agar plates and grown overnight at 37 °C. A well-separated colony was picked and grown overnight in TSB medium at 37 °C with shaking at 80 rpm. The bacterial suspension was taken from the overnight culture and inoculated with fresh TSB medium (the inoculum was 1% of the volume of the new culture). The bacteria were grown at 37 °C with shaking at 80 rpm until OD_620_ reached 0.6–0.8, after which the cells were collected by centrifugation (3500 rcf, 10 min, 20 °C).

The test was carried out in 50 mM glycine–NaOH pH 8.0, with 100 mM NaCl or without salt. The pelleted bacteria were suspended in the buffer to obtain an OD_620_ of 2.0, which corresponds to a bacterial concentration of around 10^8^ CFU/ml. Then, 100 µl of the bacterial suspension was mixed with 100 µl of the enzyme solution (final concentration 500 nM). The OD_620_ was monitored for 1 h using a spectrophotometer. The OD_620_ value at time zero was taken as 100%, and each subsequent measurement is referenced to this value. Enzyme activity is determined as the difference between normalized OD_620_ after 1 h of the control without enzyme and the sample with enzyme.

### MS analysis

Mass measurement was performed on a Waters Synapt G2 as a service at IBB PAS, Warsaw, Poland. Waters MassLynx 4.1 software was used to calculate mass from the obtained m/z values using the MaxEnt3 calculation tool.

### Cytotoxicity

#### MTT assay on HaCAT and NIH 3T3 cells

Mice fibroblasts (NIH 3T3), human keratinocytes (HaCAT), and human epithelial cells (Detroit) were cultured in DMEM medium (Gibco) supplemented with 10% FBS (Gibco or Sigma) and 1% PenStrep (Sigma). Before the experiment, cells at 80% confluency were washed with PBS (Dulbecco’s Phosphate Buffered, modified Saline, Sigma), detached using TrypLE Express (Gibco), and suspended in DMEM without phenol red (Gibco) supplemented with 10% FBS. No antibiotics were added. The density of the suspension was estimated using a benchtop cell counting system (Countess™, Invitrogen and EVE™ cell counting slides, NanoEntek) and adjusted to 5 × 10^5^ cell/ml. Cells were seeded in a 96-well plate (96-well NEST Cell Culture Plate, Polystyrene, Non-Pyrogenic) and cultured at 37 °C in 5% CO_2_ (Memmert incubator). Wells were filled with 100 µL of cell suspension. For background control, a medium without cells was used. The next day, the medium was replaced with fresh and filtered (using a 22 µM filter, Millex-GV, Merck Millipore) phenol red-free DMEM supplemented with 10% FBS with and without enzymes. After a 22 h-long treatment, 10 µl of thiazolyl blue tetrazolium bromide (MTT, POL-AURA) was added to each well to a final concentration of 0.5 mg/ml. Plates were incubated at 37 °C for an additional 3 h. Thereafter, 100 µl of dimethyl sulfoxide (DMSO, POL-AURA) was added, and plates were left for 15–45 min. Once formazan crystals dissolved, the absorbance was measured at 570 nm.

#### Toxicity assays using *G. mellonella* larvae

Final instar larvae of the greater wax moth (*G. mellonella*) were obtained from a local vendor and stored in wood shavings in the dark at 8 °C for a maximum of two weeks. One day before the experiment, larvae were transferred to 9 cm Petri dishes with a 9 cm ring of paper towel and stored overnight at room temperature in the dark. On the day of the experiment, the larvae were sorted again and batches containing > 80% healthy-looking insects > 1.5 cm in size were used for the experiments.

A total injection volume of 20 µl ± 2 µl of EL S2, EP S2, or ES S1 enzymes at a concentration of 500 nM or 5 µM in E3, 50 mM glycine buffer pH 8.0 or ultrapure (Milli-Q) water was administered by injection through the last pro-leg using a 0.5 ml 0.3 × 8 mm Micro-Fine Plus syringe (BD). For the control, untreated and needle-punctured larvae were used along with a group of larvae injected with water or glycine buffer. Enzyme-treated larvae and controls were kept at 28 °C in the dark for 7 days. Mortality was assessed daily. The numbers of larvae, cocoons, and free pupae were also recorded daily. The larvae were considered dead when they turned black or when no movement was observed, even when touched with forceps. To assess (pre-)pupal mortality, cocoons and pupae were halved at the end of the experiment, and insects with dark and watery tissues were considered dead. A group of 10 larvae was used per treatment, and the experiment was conducted on two independent occasions. At the end of the experiment, any surviving moths were killed by freezing at –20 °C for at least 1 h before disposal.

#### Toxicity assays using zebrafish

Wild type AB^OMD^ zebrafish (*Danio rerio*) line was maintained and bred at the Aquatic Facility of the Mossakowski Medical Research Institute, Polish Academy of Sciences (licenses no. 026 and 0093 from the Ministry of Science and Higher Education in Poland). Adult fish were kept in a 60 l glass tank at 26 ± 0.5 °C, with a 14:10 h day/night cycle, in water with pH 7.5 ± 0.5, conductivity 300–500 µS/cm, undetectable levels of NH_4_^+^ and NO_2_^−^. Fish were fed once or twice a day with Hikari Fancy Guppy (Hikari) dry feed. As environmental enrichment, natural gravel was used at the bottom of the tank. Additionally, a powerhead (Turbo Mini, Aquael) was installed to generate a localized water current to stimulate natural conditions. For breeding, 1.7 l breeding tanks (Tecniplast) were placed inside the glass aquaria. A piece of a black fishing net with 3 × 3 mm mesh size, partially covering the top of the breeding tank, allowed eggs to fall through while leaving the fish free to spawn naturally was used to stimulate breeding.

Due to the limited availability of purified enzymes, the acute fish embryo toxicity test (OECD test no 236) was modified as follows. Eggs were collected from naturally spawning group crosses, rinsed in E3 (standard medium used for zebrafish works (‘E3 medium (for zebrafish embryos)’, 2011), and underwent quality check. Only clutches with > 80% fertilized, healthy-looking eggs were used for experiments. Groups of 12 embryos were placed in Ø 3 cm Petri dishes containing 2 ml of E3. Before embryos reached the blastula stage, E3 was replaced with a 500 nM solution of EL S2, EP S2, or ES S1 in E3. Embryos were kept for four days in an incubator set to 28 °C, and each day the embryos were checked for malformations; dead embryos were removed, and the solution was refreshed. For control, untreated fish were maintained in E3. MS-222 was used for anesthesia during imaging and for euthanasia at the end of the experiments.

### Minimum inhibitory concentration (MIC) determination and induction of resistance development against EnpA chimeras by *E. faecalis*

*MIC determination*. The MIC of each chimeric enzyme was determined by serial dilution of protein by 1:2 in 96-well plates in 25% TSB medium with 37.5 mM glycine, pH 8.0. Exponentially growing *E. faecalis* ATCC 29212 was added to each well at a final concentration of 10^5^ CFU/well, and the plate was incubated at 37 °C for 24 h. The MIC value was determined as the lowest dose of peptidoglycan hydrolase that visibly inhibited the growth of bacteria.

*Resistance induction*. For this experiment, *E. faecalis* ATCC 29212 strain and a previously published protocol developed for bacteriolytic enzymes were used (Eichenseher et al. 2022) with the following modifications: cells were cultured with the sub-MIC concentration of each enzyme, and the MIC value was evaluated every two passages. To verify the homogeneity of bacterial culture throughout the experiment and to exclude the potential contamination with environmental bacterial strains, cultures were plated on the CHROMagar™ Mastitis GP/N plates. After 40 passages of incubation with a sub-MIC concentration, the identity of the bacteria was verified by matrix-assisted laser desorption ionization time-of-flight mass spectrometry (MALDI-TOF MS).

#### Combination of chimeras and bacteriocines

MIC for bacteriocins was determined as described above for chimeric enzymes. For synergy tests, the following range of bacteriocins was tested: MP1 0.05–6.25 µg/ml, EJ97s 0.034–25 µg/ml, nisin 0.20–25 µg/ml. MP1 was produced as previously described (Ovchinnikov et al. 2021). Synthetic peptides were used: EJ97s and garvicin KS (purchased from Pepmic (China) with > 99% purity). Nisin was purchased from Merck. Exponentially growing *E. faecalis* was added to each well of the 96-well plate at a final concentration of 10^5^ CFU/well with appropriate amounts of the antimicrobial mixtures and was incubated at 37 °C for 24 h.

## Results

### Stability

We have recently reported the generation of chimeric enzymes, in which a catalytic domain EnpA from *E*. *faecalis* was fused to one of three different SH3b domains (together with their protruding linkers) from lysostaphin (chimeric enzyme named “EL”), lysostaphin-like protein from *Staphylococcus simulans* (ES), or SpM23 peptidoglycan hydrolase from *Staphylococcus pettenkoferi* (EP). The constructed enzymes are characterized by extended tolerance to pH and ionic conditions (Mitkowski et al. 2024).

**Fig. 1:**
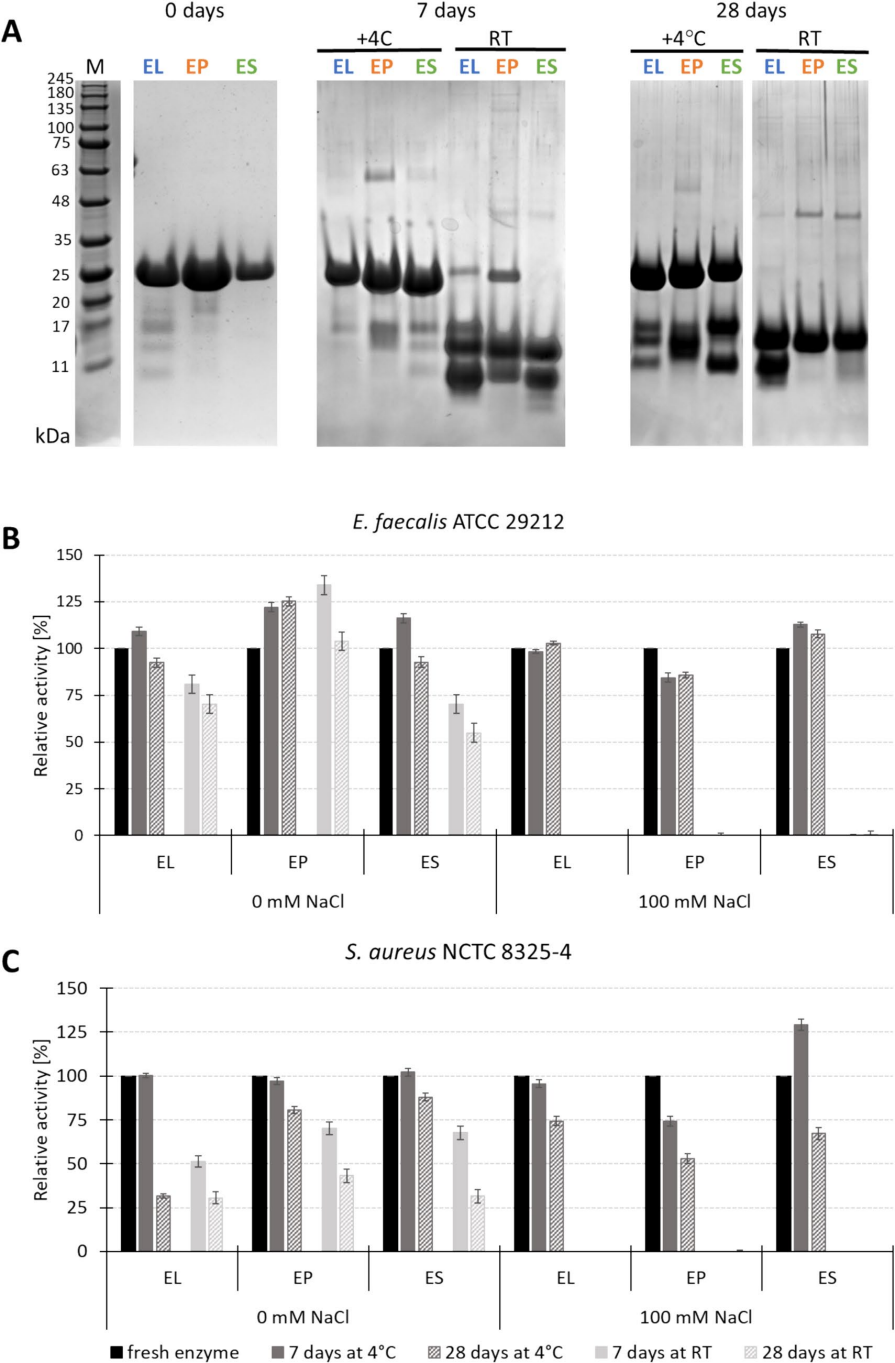
Stability and activity of the chimeras during storage in 20 mM Tris–HCl pH 8.0, 200 mM NaCl, 10% glycerol at 4 °C or room temperature (RT). **A** Protein stability monitored by SDS-PAGE. The approximate size of the chimeric enzymes: 26–27 kD, catalytic domain: 13,8 kDa, cell wall binding domains: 12 −13 kDa. **B**, **C** Activity of the enzymes against *E. faecalis* (**B**) and *S. aureus* (**C**) after 7 and 28 days of storage at + 4 °C and RT monitored in 50 mM glycine buffer, pH 8.0, and low ionic strength conditions. The activity was assessed using turbidity reduction assay and is shown as percentage of the activity of the fresh enzyme. The test was done in two biological and three technical replicates

We have observed that during storage, the chimeric enzymes degrade (Fig. 1). Electrophoretic analysis showed several additional bands, with the most abundant ones around 15 and 12 kDa, which are close to the masses of catalytic and cell wall binding domains, respectively. After 4 weeks at room temperature (RT), the band corresponding to the binding domain disappeared completely for EP and ES chimeras, while the band of around 15 kDa was still abundant in all samples.

Also, activity tests in the presence of 100 mM NaCl demonstrated decreasing activity of the enzymes over storage time (Fig. S1). To test if the enzymes are indeed degraded and split into separate but intact domains, we have run an activity test in low ionic conditions knowing that the separated catalytic domain is active only in low conductivity buffers. Activity tests performed for stored samples demonstrated that, in fact, the activity in low ionic strength conditions is still relatively high and significantly outperforms the activity detected in the presence of 100 mM NaCl (Fig. 1). These observations brought us to the conclusion that the amino acid chains most likely break in the domain-linker region, releasing separate domains.

To improve the stability of the chimera, we first tested the effect of the storage buffer with various pH levels (Fig. S2), confirming that the buffers with pH above 7 are the most suitable. We have not observed precipitation in any of the buffers tested and have not found any storage buffer that would significantly improve the proteins’ stability. Therefore, we here attempt to enhance enzyme stability by engineering their linkers, an approach that has proven successful in other chimeric proteins (Jagielska et al. 2016).

### Improving the stability of chimeras

**Fig. 2:**
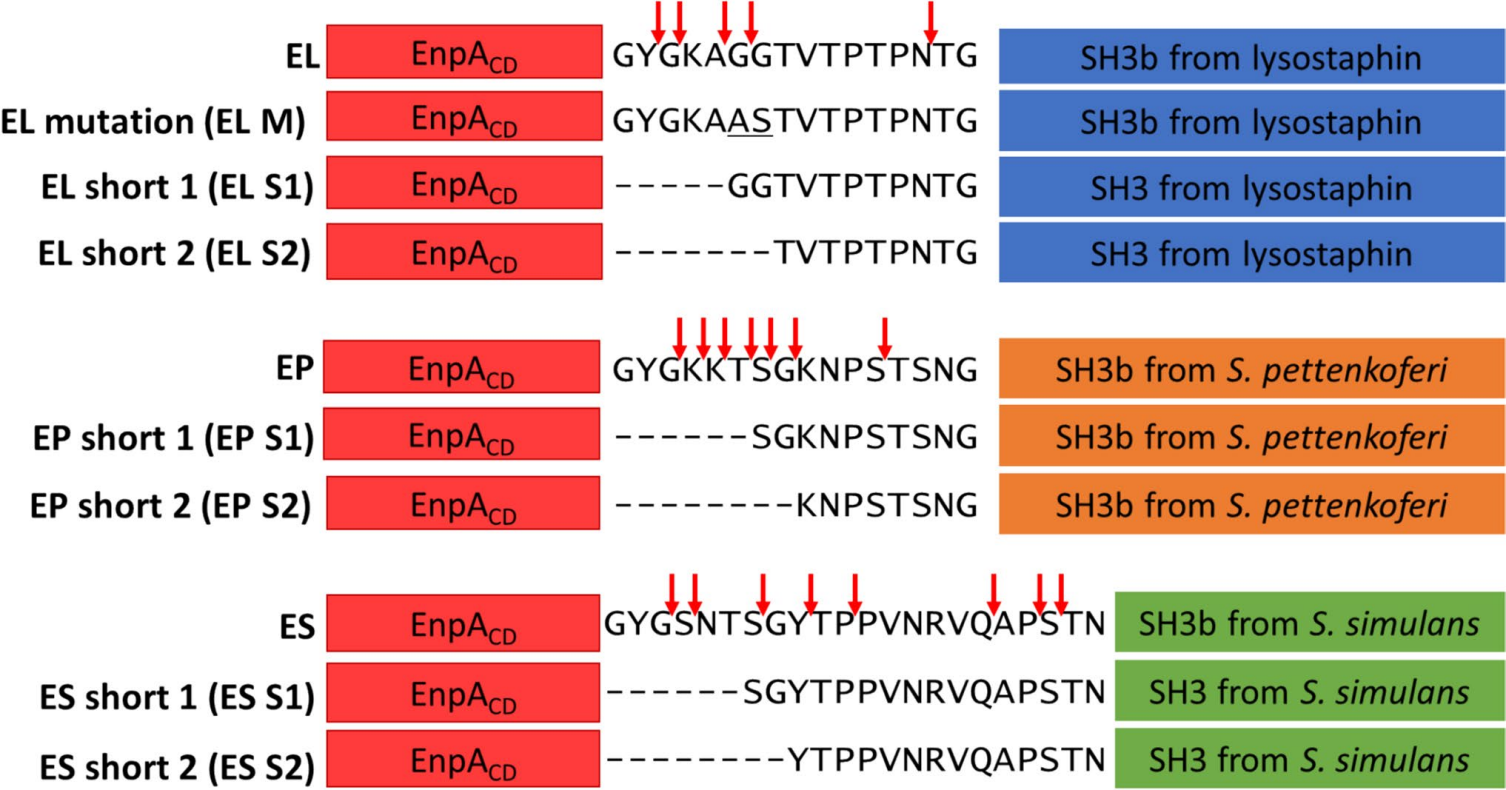
Schematic presentation of the chimeras and their variants. Red arrows indicate sites of degradation identified by MS (Fig. S3). The sign “−” means that an amino acid has been removed from a given position

To identify the position in the amino acid sequence where the chimeric enzymes break, the partially degraded protein samples were analysed by mass spectrometry (MS), and the FindPept2 tool (Gattiker et al. 2002) was used for fragment mass analysis. Several putative cleavage sites within the linkers were identified. The results of MS analysis confirmed that the chimera breaks down into two domains and that the linker is the most fragile fragment (Fig. S3). In general, the N-terminal parts of the linkers were more prone to degradation, and some of the putative cutting sites were next to or close to glycines (Fig. 2).

Based on MS analysis, we decided to shorten the linkers by removing the first 5 or 8 amino acids, as multiple cleavage sites were identified within this region. Furthermore, two glycines (GG) in the linker of the chimera EL were substituted by alanine and serine (AS) to avoid putative self-cleavage, as the EnpA can cut glycyl-glycine bonds (Reste De Roca et al. 2010; Małecki et al. 2021; Mitkowski et al. 2024).

In order to simplify chimeric protein purification, the step of His-tag cleavage by TEV protease was omitted. Prior to this, we used a turbidity reduction assay to test whether the N-terminal His-tag affected the enzyme’s activity. This assay showed that the His-tag sequence only slightly affected the activity of the chimeras in low ionic strength conditions, while in the presence of salt, the activity of all chimeric enzymes was similarly reduced. Therefore, such constructs could be used for relative comparisons (Fig. S4).

**Fig. 3:**
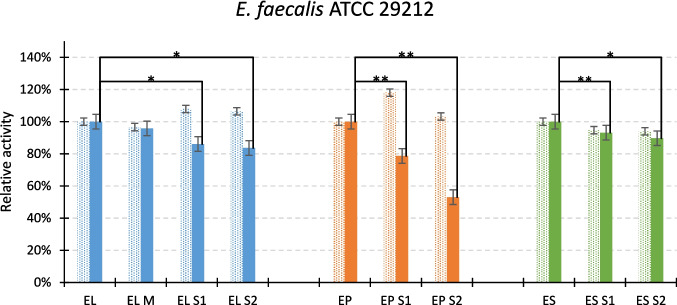
Activity of chimeras and their variants against *E. faecalis* ATCC 29212 determined by a turbidity reduction assay. Dotted bars indicate activity measured in low ionic strength (glycine buffer without salt), while solid bars in the presence of 100 mM NaCl. The activity of the initial versions of chimeras served as reference (100%). The graph shows the mean value with the standard deviation from two and six biological replications (for low and high ionic strength, respectively) and in two technical replications. Statistical analysis—ANOVA with Tuckey’s post hoc test

First, we checked whether modifications of the linkers influence the activity of the generated variants on three different bacterial species: *E. faecalis* (Fig. 3), *Staphylococcus aureus*, and *S. simulans* (Fig. S5). The level of activity was the same as for the initial chimeras in low ionic strength conditions. Some differences were noted when the reaction was performed in the presence of salt. In general, in 100 mM NaCl conditions, shortening of the linker caused a decrease in the activity of the enzymes tested against *E. faecalis*, with the exception of the EL M variant (Fig. 2). In the case of *S. simulans,* shorter ES chimeras (ES S1, ES S2) showed higher activity in the presence of 100 mM NaCl as compared to ES (Fig. S5).

The activity test was then performed on a larger number of species (Table S1). In the vast majority of cases, the activities of the modified variants were at the same level as the activity of the initial enzymes (Fig. S6).

**Fig. 4:**
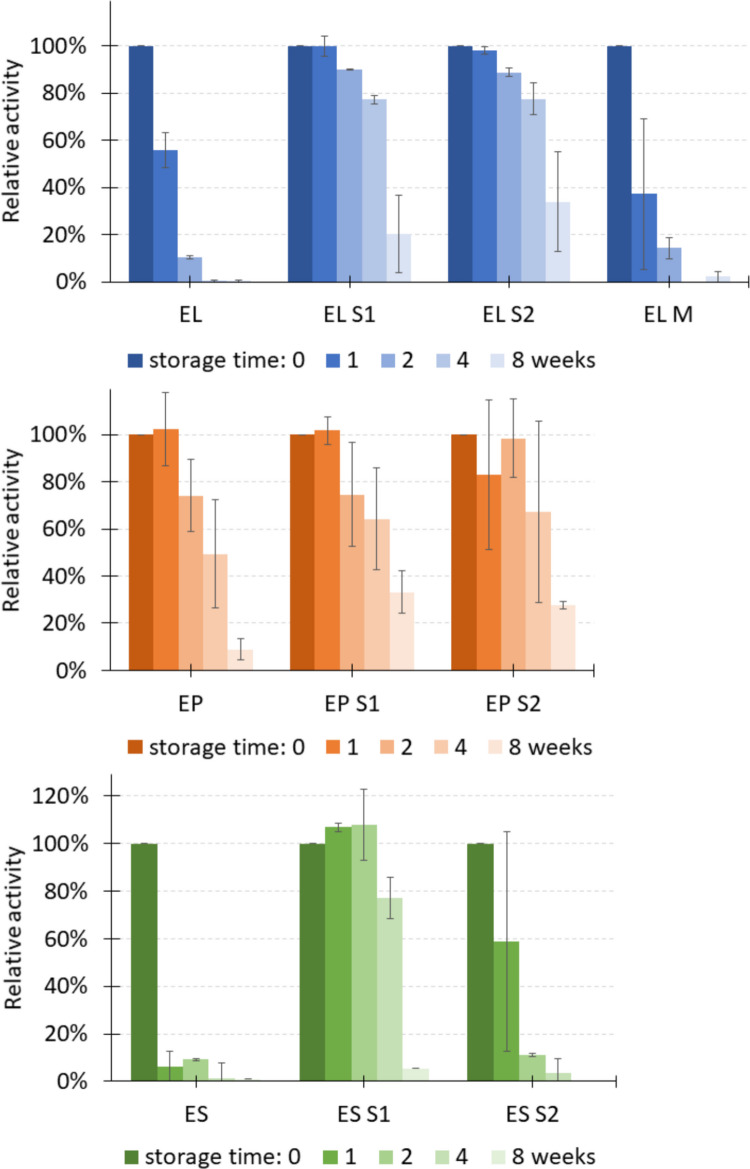
Activity of chimeras with modified linker after storage at room temperature against *E. faecalis* ATCC 29212 determined by a turbidity reduction assay. The experiment was performed in two biological repetitions, each in two technical repetitions, the graph shows mean activity of a chimera variant referring to unmodified enzyme activity on day 0; the calculated SD is included in the graph

In the next step, the effect of introduced modifications on the stability of variants was evaluated. The enzymes were stored in 50 mM HEPES pH 7.5 buffer, 400 mM NaCl, and 10% glycerol at 4 °C, room temperature, and 37 °C. The activity of the stored variants was examined after 1, 2, 4, and 8 weeks and compared to the activity on day 0 (Fig. 4 and Fig. S7). No improvement in stability was observed for EP variants independent of the storage temperature; however, it is worth underlining that the stability of the EP initial variant was relatively high, and it was the best among the three initial chimeras. On the other hand, extended stability was clearly demonstrated for both EL and ES variants with shortened linkers, but not for the variant with double substitution (EL M). A similar effect was observed when the chimeras were stored at 4 °C or room temperature; however, in the latter case, the enzymes remained active for only one month, and the ES S2 chimera was losing its activity even faster (Fig. 4). In summary, the most pronounced improvement in stability was achieved in the chimeras EL S1, EL S2, and ES S1.

#### Safety

While engineering bacteriolytic enzymes for potential applications, potential toxicity must be considered. We performed three different toxicity assays to verify whether the enzymes triggered any adverse effects on eukaryotic cells *in vitro* and organisms *in vivo*. For these experiments, the most stable variants were used, namely, EL S2, EP S2, and ES S1.

**Fig. 5:**
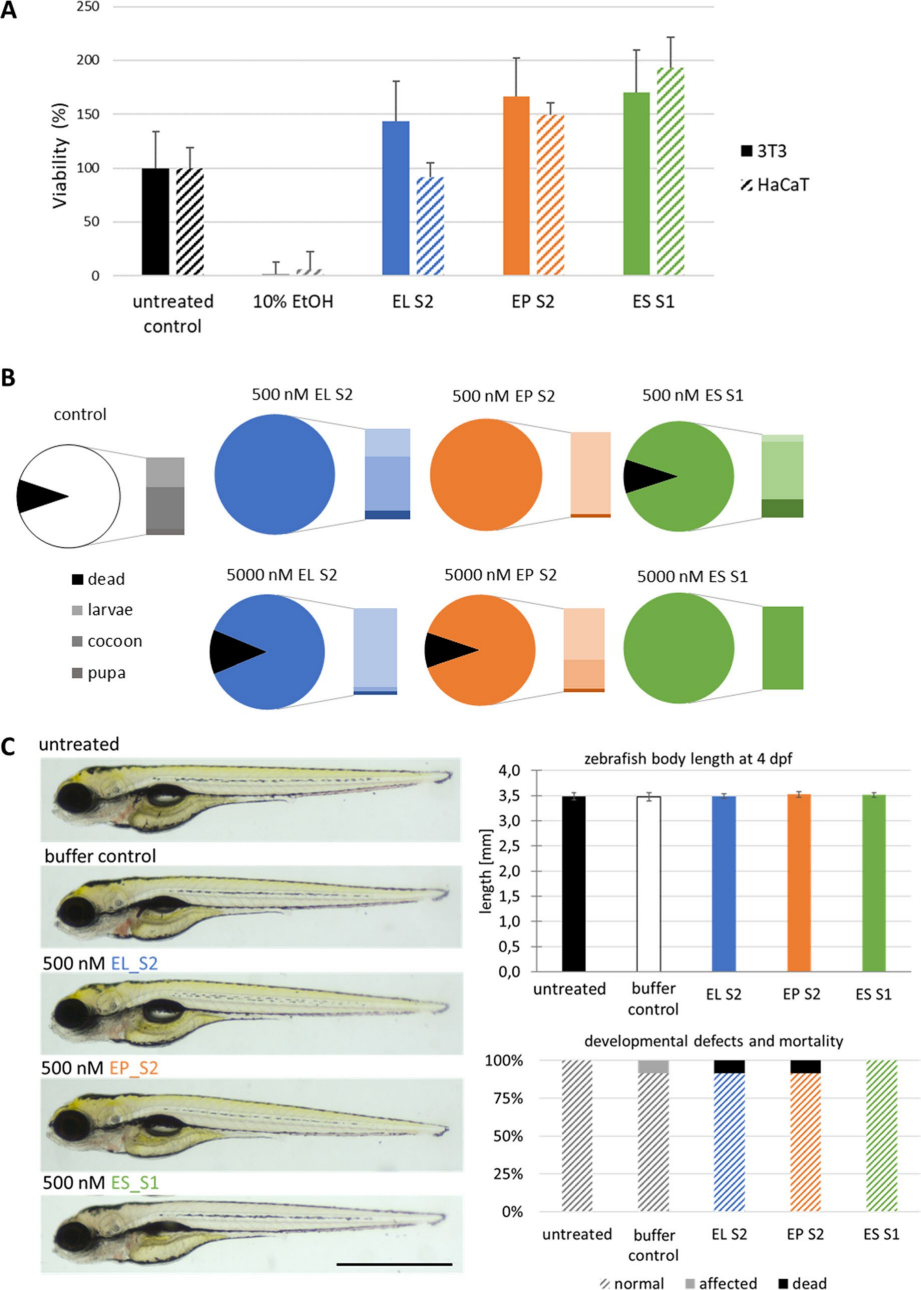
Engineered chimeras cytotoxicity assessment. **A** MTT assay was used to examine the metabolic activity of HaCAT human keratinocytes and NIH 3T3 mouse fibroblast cells exposed for 24 h to a 500 nM concentration of enzymes. Ethanol was used as a known control triggering toxicity. The graph represents a summary of three independent experiments, each performed in nine technical repetitions. **B** Distinct aspects of *G. mellonella* development and survival in response to injection with different concentrations of enzyme solution. The pie chart displays the proportions of life vs. dead larvae, and the bar chart shows the proportion of live *G. mellonella* at different developmental stages. **C** An example of the Fish Embryo Acute Toxicity (FET) Test. Zebrafish embryos were exposed by immersion to a 500 nM concentration of EL S2, EP S2, or ES S1 enzymes in E3 medium. Equal concentration of protein storage buffer was used for additional control (buffer control). Left panel, lateral view of the zebrafish larvae at 96 hpf showing normal morphology. Top right panel, bar plot showing body length at 96 hpf as one of the indicators of proper zebrafish growth. Bottom right panel, stacked bar chart showing proportions of different phenotypes (normal, affected, dead). *n* = 12, error bar represents SD. The experiment was repeated three times with similar results. Scale = 1 mm

The cytotoxicity of EL S2, EP S2, and ES S1 was assessed *in vitro* with a widely used colorimetric assay of cell metabolic activity (MTT assay). In this assay, all tested chimeras showed no cytotoxic effects, as evidenced by the absence of any reduction in cell metabolic activity. An increase in metabolic activity was even observed when mouse fibroblasts (NIH 3T3) and human keratinocytes (HaCAT) were exposed to 500 nM chimeras for 24 h (Fig. 5A). Similarly, enzymes applied at a concentration of 500 nM or 5 µM to Detroit cells had no adverse effect on their metabolic activity (Fig. S8). In the control, where cells were exposed to 10% ethanol, treatment affected cell survival and metabolic activity as expected, proving that cells were sensitive to toxic treatments (Fig. 5A).

Next, the toxicity of the enzymes was tested on living organisms. In adherence to the 3R principles (animal use Replacement, Reduction, and Refinement), we chose to perform the toxicity testing with non-mammalian models, starting from the greater wax moth (*Galleria mellonella*) larvae. When exposed to toxic compounds, *G. mellonella* larvae activate their immune system, produce melanin (melanization), and turn black (Dinh et al. 2021; Serrano et al. 2023). When assessing the basic health status of *G. mellonella* beside melanization, the survival rate, mobility, and the progress in metamorphosis were also scored (Serrano et al. 2023). Throughout the 7-day observation period, no significant difference in survival or progress of morphogenesis was observed among *G. mellonella* larvae injected with 500 nM or 5 µM solutions of tested chimera, compared to the mock control group (Fig. 5B). Also, the pigmentation of the larvae injected with enzymes did not differ from mock-injected control (data not shown), suggesting that EL S2, EP S2, and ES S1 are non-toxic to invertebrate organisms.

To strengthen these findings, we extended our toxicity assessment to zebrafish (*Danio rerio*). We found that zebrafish embryos exposed to 500 nM of tested chimeras showed normal development (Fig. 5C). No signs of coagulation or problems with somite formation, tail detachment, or altered heartbeat were observed throughout the treatment period (4–96 hpf, hours post fertilization) (data not shown). The fish displayed normal morphology, pigmentation, and did not develop oedema. In addition, enzymes at a concentration of 500 nM did not affect survival (Fig. 5C). Therefore, the results of the acute developmental toxicity test on zebrafish embryos, combined with findings from *G. mellonella*, consistently indicate that the tested chimeras can be considered non-toxic and non-teratogenic.

## MIC determination and resistance development

The most demanded features for new antimicrobial compounds are low MIC and low prevalence of resistance development. Generated chimeras were investigated also in this respect. MIC values were determined for the best-performing and most stable variants, namely EL S2, EP S2, and ES S1 (Table 2).

**Table 2:** MIC values determined for selected enzymes variants and bacteriocins

	MIC value [µg/ml]		
	***S. aureus*** ** NCTC 8325-4**	***E. faecalis*** ** ATCC 29212**	***E. faecalis*** ** ATCC 29212 EL_res**
**EL S2**	3.12	3.12	> 100
**EP S2**	12.50	6.25	12.50
**ES S1**	3.12	3.12	6.24
**Nisin**	1.56	12.50	12.50
**MP1**	12.50	6.25	6.25
**EJ97s**	6.25	0.78	3.12
**Garvicin KS**	3.12	1.56	1.56

**Fig. 6:**
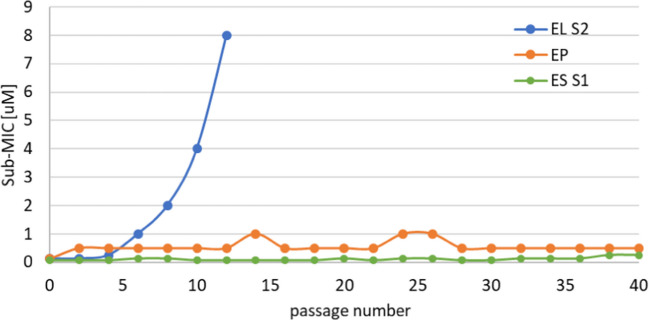
Generation of resistance. To test the prevalence of resistance development *E. faecalis* cells were grown in the presence of sub-MIC concentrations of EL S2, EP S2, or ES S1 chimeras

To trigger induction of resistance development, *E. faecalis* was cultured in the presence of chimeras at sub-MIC concentrations for 40 passages. For EP S2 and ES S1 chimeras, we observed only a slight increase in MIC after two passages, but it remained at a relatively constant level even after 40 passages (Fig. 6). The MS analysis performed at the end of the experiment confirmed the identity of the bacteria used during the experiment.

In the case of EL S2, a significant increase in MIC value was observed already after 4 passages, reaching over 30-fold MIC change after 10 passages in the presence of the enzyme at the sub-MIC concentration. The resistance of the strain was confirmed in a separate MIC determination experiment, and MS was used to confirm that it was *E. faecalis* and not any contamination. Interestingly, the EL resistant strain was still susceptible to ES and EP chimeras, and the MIC values for these two enzymes were only slightly increased.

## Combination with bacteriocins

We recently demonstrated that a combination of bacteriolytic enzyme and bacteriocins (antimicrobial peptides from bacteria) was efficient in eliminating a range of Gram-positive pathogens (Kranjec et al. 2024). We therefore checked if the combination of chimeric enzymes generated here and bacteriocins could enhance the antimicrobial effects. For these tests, we chose four bacteriocins with different mechanisms of action targeting *E. faecalis* and other bacteria. Nisin is known to target cell membranes and inhibit cell wall synthesis; garvicin KS (Ovchinnikov et al. 2016) targets the cell membrane; micrococcin P1 (Su, TL, 1948) inhibits translation, while EJ97s (Gálvez et al. 1998; Ovchinnikov et al. 2022) targets the membrane protein RseP and kills cells by a hitherto unknown mechanism (Kranjec et al. 2021; Field et al. 2023). The MIC values were determined for nisin, MP1, EJ94s, and garvicin KS alone (Table 2) as well as in combination with ES S1 against *E. faecalis* to assess their potential synergistic interaction (Table S2). However, only additive effects between the bacteriocins and ES S1 were observed. Furthermore, garvicin KS was also tested together with EL S2, but only additive effects were observed. Thus, no clear synergistic or antagonistic effects were detected in the bacteriocin-enzyme combinations tested (Table S2).

We also tested the susceptibility of the EL S2 resistant variant, generated in the experiment described above, to these four bacteriocins. The MIC value for nisin, micrococcin P1, and garvicin KS was not changed, while for EJ97’s bacteriocin, the MIC value increased approximately four-fold (Table 2).

Thus, although bacteriocins did not act synergistically, they can be used in combination with bacteriolytic enzymes to limit the possible effect of resistance development.

## Discussion

With the worldwide health crisis caused by the spread of antibiotic resistance, bacteriolytic enzymes, which have a precisely defined antimicrobial specificity, a distinct mode of action, and a low prevalence of resistance development, are getting increasing attention as an attractive alternative to antibiotics (Fernandes and Jobby 2022; Kushwaha et al. 2024; Sisson et al. 2024).

Peptidoglycan hydrolases produced by phages or bacteria are often not naturally optimized to meet the stringent requirements of antimicrobial applications, necessitating engineering to enhance their efficacy and suitability for prevention and treatment (Osipovitch and Griswold 2015; Jagielska et al. 2016; Verbree et al. 2018; Eichenseher et al. 2022; Kaus-Drobek et al. 2025). While potent bacteriolytic activity and appropriate specificity are critical attributes, they alone do not guarantee the practicality of these enzymes as antimicrobial agents. To be effective, such enzymes must not only eliminate bacteria but also retain their activity under complex biological media and be safe for humans and animals. Furthermore, stability is essential to ensure adequate shelf life during storage and a sufficient half-life during treatment. Learning from the rapid development of resistance to antibiotics, next-generation antimicrobials should also demonstrate a low propensity for resistance development to ensure sustained efficacy.

The modular architecture of peptidoglycan hydrolases enables relatively straightforward engineering of the enzymes through the shuffling and fusion of various domains. Such chimeric enzymes have been shown to acquire broader tolerance to ionic strength and pH values (Jagielska et al. 2016; Mitkowski et al. 2024; Kaus-Drobek et al. 2025), exhibit increased activity (Yang et al. 2015; Yang et al. 2017; Vázquez et al. 2017), display enhanced activity toward a wider range of bacterial species (Yang et al. 2015), or possess an extended half-life in the body (Seijsing et al. 2018). Modifications aimed at improving enzyme properties may involve more precise alterations, such as manipulation of surface charge, as demonstrated in the bacteriophage Cpl-7 protein, resulting in improved enzyme activity (Diez-Martínez et al. 2013). Peptidoglycan hydrolases typically comprise structurally well-defined domains with distinct functions, connected by a linker. The linker plays a crucial role by influencing the structural flexibility of multidomain proteins, providing adequate space between domains to prevent interference, facilitate proper folding, and control the enzyme activity (Pohane et al. 2015; Oechslin et al. 2021; Oechslin et al. 2022). Various characteristics of the linker, including its length, amino acid composition, hydrophobicity, and secondary structures, contribute to its functionality (Pohane et al. 2015; Eichenseher et al. 2022; Carratalá et al. 2023).

Recently, we reported the generation of new chimeric enzymes consisting of the M23 catalytic domain from the enterococcal EnpA protein fused with one of three different SH3b binding domains, each with its native linker. This fusion significantly increased the enzyme’s tolerance to ionic and pH conditions (Mitkowski et al. 2024). Similar effects were observed in chimeric enzymes created by fusing the M23 catalytic domain from the staphylococcal LytM autolysin with the SH3b binding domain from lysostaphin. However, in both cases, degradation of the chimeric enzymes into separate domains was observed, and further engineering of the linker was essential to improve enzyme stability (Jagielska et al. 2016). Aside from enhanced stability, modifications to the linker can lead to accelerated activity of the chimeras. ClyJ endolysin variants with modified linker length demonstrated this effect, with the chimera featuring the shortest linker exhibiting increased bacteriolytic activity, improved thermostability, broader specificity, and reduced cytotoxicity, confirmed in a mouse model. The authors hypothesize that shortening the linker could increase the rigidity of the molecule, thereby accelerating binding to the substrate. Alternatively, the appropriate linker length could allow for simultaneous binding and catalytic activity by ensuring the proper separation of both domains (Yang et al. 2020). Similarly, shortening of the linker in the SA.100 chimeric enzyme targeting staphylococci led to significant improvement of enzyme performance, while the XZ.700 derivative displayed lower thermal stability. The effect was attributed to the optimized arrangement of two adjacent functional domains, which dictates their alignment with the corresponding cleavage or binding sites within the substrate (peptidoglycan) (Eichenseher et al. 2022). Cleavage of linkers also was reported for native, multidomain endolysins, and such protein processing was postulated as one of the mechanisms regulating enzyme activity (Oechslin et al. 2021; Oechslin et al. 2022).

Safety of new antimicrobials is one of the obvious demands when biomedical applications are considered. Many of the bacteriolytic enzymes have been evaluated in this respect using viability assays of eukaryotic cell lines, commonly fibroblasts and keratinocytes (Wang et al. 2023). In our laboratory, in addition to the standard MTT assay, we have used two additional cost- and time-effective assays for bacteriolytic enzymes’ toxicity evaluation.

The *G. mellonella* model is primarily used for infectious studies, but recently, *G. mellonella* is more often employed to assess the effectiveness and potential toxicity of new antibacterial drugs (Serrano et al. 2023). *G. mellonella* larvae are easy to handle and provide quick insights through parameters like melanization and survival, making them ideal for preliminary assessments. *Danio rerio*, a vertebrate model, is frequently used in biomedical studies also for drug discovery. This approach followed guidelines from OECD test no. 236, which focuses on evaluating acute toxicity in fish embryos. Zebrafish embryos are particularly advantageous because they allow for the observation of early developmental stages in a transparent, rapidly growing system, providing insights into potential teratogenic and toxic effects. Zebrafish embryos, with their genetic similarity to humans, fast development, and transparent body, enable detailed, real-time observations of systemic and developmental toxicity. Both insect and fish models nicely complement *in vitro* models and reduce reliance on mammalian testing while offering complementary and efficient toxicity evaluation. Our results from both *in vitro* and *in vivo* assays support the safety profile of all three enzymes (EL S2, EP S2, and ES S1) with bacteriolytic activity, highlighting their potential as safe and promising candidates for further development for pharmaceutical or agricultural applications. To date, no side effects of endolysins treatment in animal models or on humans (volunteers) have been reported (Jun et al. 2014; Jun et al. 2017), thereby strongly supporting the safety of enzybiotic-based drug treatments (Liu et al. 2023).

Resistance development is the main concern not only in medicine but also in the veterinary and food industries. In general, peptidoglycan hydrolases are considered antimicrobials with a very low prevalence of resistance development; however, there are surprisingly few experimental data addressing this issue. Antistaphylococcal engineered enzymes SA.100 and its modified version XZ.700 were reported not to develop resistance in *in vitro* assays. Moreover, resistance was not found among *S. aureus* isolates obtained from a patient treated with the same enzyme for several months (Totté et al. 2017). Endolysins have been suggested to be refractory to resistance development in bacteria due to their highly conserved binding and cleavage sites in peptidoglycan (Schmelcher et al. 2012). Moreover, the fact that investigated chimeras have two catalytic domains targeting different bonds in PG could also lower the probability of resistance development. This is also in agreement with previous research showing that the rate of resistance development against staphylococcal PGHs decreases with an increasing number of different catalytic domains within the same enzyme (Becker et al. 2016).

In this work, resistance development was observed for the EL chimera, but not the other variants. Lysostaphin, a bacteriocin produced by *S. simulans*, was shown to trigger resistance development, and various *S. aureus* mutant strains featuring alterations within the pentaglycine bridge and reduced susceptibility to lysostaphin have been described (Gründling et al. 2006). This can be explained by the fact that the mechanism of lysostaphin resistance exists in nature and is caused by the substitution of glycines to serines in the cross bridges of peptidoglycan (Dehart et al. 1995). Such changes might affect both catalytic activity as well as cell wall binding by the SH3b domain. This domain has been shown to directly bind pentaglycine cross bridges, and alterations, like the replacement of glycine with serine, abolished domain binding (Mitkowski et al. 2019).

Our experiments demonstrated that peptidoglycan hydrolases, when used as bacteriolytic enzymes, can potentially trigger the development of resistance, as observed in the case of our chimeric enzyme EL S2. However, we did not observe such effects in two other chimeras, despite all tested enzymes sharing the same catalytic domain. This finding highlights the importance of experimentally assessing resistance development for each bacteriolytic enzyme individually. We plan to continue our investigations to uncover the underlying mechanism of the observed resistance. At the same time, the EL S2 resistant variant remained susceptible to two other chimeric enzymes and three out of the four tested bacteriocins, further supporting the rationale for using combinations of antimicrobials with different modes of action to mitigate the risk of resistance development.

In summary, we report here a strategy for the stabilization of peptidoglycan hydrolase chimeras by linker modification designed based on HPLC–MS analysis. We have also shown that our enzymes do not display any cytotoxic effects and can be combined with other natural antimicrobials, like bacteriocins. Most importantly, we have tested the prevalence of resistance development and demonstrated that, although bacteriolytic enzymes are generally considered not to trigger resistance development, such statements should always be confirmed experimentally.

## Supplementary Information

Below is the link to the electronic supplementary material.

ESM1 (DOCX 1.42 MB)

## Data Availability

Data are available from the authors upon request.

## Materials availability

Available upon request from the authors.
