# Supplementary material for: Enhancing stability and safety of chimeric peptidoglycan hydrolases by linker engineering
Source: Appl Microbiol Biotechnol. 2026 Jan 12;110:21. doi: 10.1007/s00253-025-13651-7 (PMC12799751; doi:10.1007/s00253-025-13651-7)
Supplement: Supplementary file 1 [file 253_2025_13651_MOESM1_ESM.docx]

**Supplementary materials**

**Figure S1**. Lytic activity of chimeras EL, EP, ES stored at 4°C and 22°C. The activity of the stored proteins was tested after 7 and 28 days in turbidity reduction assay on *S. aureus* (A) and *E. faecalis* cells (B).

**Figure S2.** The effect of buffer pH on stability of chimeric enzymes during storage. Activity of enzymes stored at room temperatures for 28 days in different buffers: citric acid pH 3.2, Na+/K+ phosphate pH 5.0, ADA pH 6.5, HEPES pH 7.5, Tris-HCl pH 8.0, Tris-HCl pH 8.5, CAPS pH 10.0 presented as the percentage of the control (protein stored at -80°C). The activity was evaluated using turbidity reduction assay against *E. faecalis* ATCC 29212 in 50 mM glycine buffer pH 8.0 without or with 100 mM NaCl and the final concentration of the enzymes tested was 500 nM. The results are presented as the % of the OD_600_ reduction compared to the control without enzyme.

**Figure S3.** Electrospray ionization mass spectrometry (ESI-MS) analysis of chimeras’ degradation products. The image shows a fragment of the linker sequence of each enzyme with the breakdown sites marked by black lines and the mass corresponding to a given fragment. In the graphs, red squares indicate the masses to which the corresponding enzyme fragments were matched.

**Figure S4**. The effect of His-tag on activity of chimeric enzymes. The activity of the generated chimeras with and without His-tag was tested in turbidity reduction assay in 50mM glycine buffer, pH 8.0 and 500nM enzymes as described in Materials and Methods section and is presented as percent of reduction of the initial OD_600_ after 1 hour incubation.

**Figure S5**. The effect of linker modification on activity of chimeric enzymes. The activity was monitored in turbidity reduction assay for 60 minutes and is presented as the percentage of the activity of the initial versions of chimeras that served as reference (100%). Dotted bars indicate activity measured in low ionic strength (glycine buffer without salt), while solid bars in the presence of 100 mM NaCl. The experiment was done in triplicates and repeated - twice for *S. aureus* and three times for *S. simulans*. Statistical analysis - ANOVA with Tuckey's post hoc test.

| **Table S1.** List of bacterial strains used in evaluation of activity of EL, ES, EP variants (Figure S6). | | | | |
| --- | --- | --- | --- | --- |
| **Bacteria type** |  | **PG chemotype** | |  |
|  | **Cross-bridge composition** | **Schleifer and Kandler (1972)** | **Schumann (2011)** | **Bacterial strain** |
| Gram-negative | direct | A1γ | A31 | *Pseudomonas aeruginosa* DSMZ 939 |
|  |  |  |  | *Kleibsella pneumoniae* DSMZ 789 |
|  |  |  |  | *Escherichia coli* DSMZ 1103 |
|  |  |  |  | *Yersinia ruckeri* CCM 4620 |
| Gram-positive | direct | A1α | A11 | *Bacillus subtilis* DSMZ 10 |
|  |  |  |  | *Corynebacterium striatum* DSMZ 20668 |
|  |  |  |  | *Listeria monocytogenes* DSMZ 15675 |
|  | L-Ala-L-Ala | A3α | A11.5 | *Streptococcus equi* subsp. *zooepidemicus* CCM 7316 |
|  |  |  |  | *Streptococcus uberis* DSMZ 20569 |
|  |  |  |  | *Streptococcus pyogenes* CCM 7418 |
|  |  |  |  | Streptococcus dysgalactiae DSMZ 20662 |
|  |  |  |  | *Streptococcus salivarius* DSMZ 20569 |
|  |  |  |  | *Streptococcus agalactiae* DSMZ 6784 |
|  | L-Ala-L-Ala-L-Ala | A3α | A11.5 | *Streptococcus salivarius s*ubsp. *thermophilus* DSMZ 20259 |
|  | D-Asp | A4α | A11.31 | *Lactococcus lactis* CCM 1877 |
|  |  |  |  | *Enterococcus faecium* DSMZ 2146 |
|  |  |  |  | *Lactobacillus paracasei* DSMZ 5622 |
|  | Ala-Gly-Gly-Gly-Gly-Gly | A3α | A11.2 | *Staphylococcus lentus* PCM 2441 |
|  | Ala-Gly-Gly-L-Ser-Gly-Gly | A3α | A11.3 | S*taphylococcus epidermidis* ATCC 12228 |
|  | L-Thr-Gly | A3α | A11.24 | *Streptococcus canis* DSMZ 20715 |
|  | L-Ala-D-Glu(Gly)-L-Lys-D-Ala | A2 | A11.pep | *Micrococcus luteus* ATCC 10240 |
|  | L-DAP-Gly | A3γ | A41.1 | *Cutibacterium acnes* PCM 2334 |

**Figure S6**. Activity of enzyme variants on collection of bacterial strains (See Table S1). The turbidity reduction assays were run for one hour using 1 µM enzyme in 50 mM glycine buffer, pH 8.0, 100nM NaCl.

4°C

Relative activity %

|  | 0W | 1W | 2W | 4W | 8W |
| --- | --- | --- | --- | --- | --- |
| EL | 100 | 100.231 | 102.17 | 102.428 | 6.90967 |
| EL M | 100 | 99.6265 | 104.11 | 95.1068 | 63.4686 |
| EL S1 | 100 | 90.4163 | 99.0055 | 101.149 | 83.9294 |
| EL S2 | 100 | 104.502 | 99.6074 | 90.2674 | 100.878 |

Relative activity %

|  | 0W | 1W | 2W | 4W | 8W |
| --- | --- | --- | --- | --- | --- |
| ES | 100 | 100.748 | 102.939 | 95.8596 | 22.764 |
| ES S1 | 100 | 102.151 | 103.64 | 108.903 | 105.444 |
| ES S2 | 100 | 98.9247 | 108.276 | 99.2925 | 103.552 |

Relative activity %

|  | 0W | 1W | 2W | 4W | 8W |
| --- | --- | --- | --- | --- | --- |
| EP | 100 | 99.0401 | 101.879 | 91.6016 | 80.2507 |
| EP S1 | 100 | 119.879 | 94.8092 | 81.6208 | 71.4071 |
| EP S2 | 100 | 100.866 | 96.7617 | 99.97 | 60.3086 |

37°C

Relative activity %

|  | 0W | 1W | ND | 4W | 8W |
| --- | --- | --- | --- | --- | --- |
| EL | 100 | 77.2879 | 0 | 18.2449 | 2.12213 |
| EL M | 100 | 47.2489 |  | 34.948 | 3.79101 |
| EL S1 | 100 | 76.9633 |  | 31.1149 | -3.3806 |
| EL S2 | 100 | 54.4121 |  | 44.9535 | 3.03554 |

Relative activity %

|  | 0W | 1W | ND | 4W | 8W |
| --- | --- | --- | --- | --- | --- |
| EP | 100 | 49.5103 | 0 | 17.5475 | 1.05265 |
| EP S1 | 100 | 74.7845 |  | 31.606 | 8.1312 |
| EP S2 | 100 | 102.282 |  | 46.8683 | 43.7436 |

Relative activity %

|  | 0W | 1W | ND | 4W | 8W |
| --- | --- | --- | --- | --- | --- |
| ES | 100 | 49.6868 | 0 | 6.26971 | 4.55437 |
| ES S1 | 100 | 53.4637 |  | 12.7684 | 5.2179 |
| ES S2 | 100 | 44.5216 |  | 51.7228 | -1.224 |

**Figure S7**. Activity of the variants monitored during storage at 4°C and 37°C for up to 8 weeks (W). The proteins were stored in the indicated conditions and the activity tested in turbidity reduction assay with 0.5 µM enzymes in 50mM glycine pH 8.0 100mM NaCl and 60 min treatment of *E. faecalis* cells in RT.

**Figure S8**. MTT assay results for the Detroit cell line. Detroit cells were seeded at a starting density of 5×10³ cells per well, let to grow for one day, and treated for 24 hours with selected chimeras at concentrations of 100 nM or 500 nM in DMEM supplemented with 10% FBS (full medium). Cells cultured in full medium alone served as the negative control (set as 100% viability), while full medium containing 10% ethanol was used as the positive control due to its well-established cytotoxicity. Results are presented as a percentage of viable cells relative to the control. EL treatment at both concentrations maintained cell viability comparable to the untreated control, indicating low or no cytotoxicity. EP and ES treatments resulted in a slight reduction in viability; however, cell viability remained above 80–90%, a range generally considered non-cytotoxic in MTT assay guidelines. Based on these criteria, none of the tested chimeras exhibited significant cytotoxic effects under the applied conditions, while the ethanol control produced a marked decrease in viability, confirming the assay's responsiveness.

| **Table S2**. Values of FIC determined for combinations of selected enzyme variants and bacteriocins. | | |
| --- | --- | --- |
|  | FIC VALUE | |
|  | *E. faecalis* DSMZ 20376 | *S. aureus* NCTC 8325-4 |
| EL S2 VS GARVICIN KS | 0,52 | 0,52 |
| EL S2 VS ES S1 | 0,52 | 0,52 |
| ES S1 VS GARVICIN KS | 0,52 | 0,52 |
| ES S1 vs MP1 | 0,52 | nd |
| ES S1 vs EJ97s | 0,51 | nd |
| ES S1 vs nisin | 0,51 | nd |
